# Functional packaging of seeds

**DOI:** 10.1111/nph.17299

**Published:** 2021-04-03

**Authors:** Jessica C. Huss, Notburga Gierlinger

**Affiliations:** ^1^ Department of Nanobiotechnology Institute of Biophysics University of Natural Resources and Life Sciences (BOKU) Vienna Muthgasse 11/II Vienna 1900 Austria

**Keywords:** diaspore adaptations, hard plant shells, physical dormancy, sclerenchyma, seed storage and protection, serotiny, tissue mechanics

## Abstract

The encapsulation of seeds in hard coats and fruit walls (pericarp layers) fulfils protective and dispersal functions in many plant families. In angiosperms, packaging structures possess a remarkable range of different morphologies and functionalities, as illustrated by thermo and hygro‐responsive seed pods and appendages, as well as mechanically strong and water‐impermeable shells. Key to these different functionalities are characteristic structural arrangements and chemical modifications of the underlying sclerenchymatous tissues. Although many ecological aspects of hard seed encapsulation have been well documented, a detailed understanding of the relationship between tissue structure and function only recently started to emerge, especially in the context of environmentally driven fruit opening and seed dispersal (responsive encapsulations) and the outstanding durability of some seed coats and indehiscent fruits (static encapsulations). In this review, we focus on the tissue properties of these two systems, with particular consideration of water interactions, mechanical resistance, and force generation. Common principles, as well as unique adaptations, are discussed in different plant species. Understanding how plants integrate a broad range of functions and properties for seed protection during storage and dispersal plays a central role for seed conservation, population dynamics, and plant‐based material developments.

## Introduction

During the course of plant evolution, fundamental changes in plant reproduction occurred in the Devonian (Gerrienne *et al*., 2004; Bonacorsi *et al*., 2020), along with major diversification periods in the Cretaceous (van der Kooi & Ollerton, 2020), that paved the way for the widespread seed habit. Today, there are an estimated *c*. 420 000 species of seed plants (Govaerts, 2003) that produce seeds and larger dispersal units in the range of approximately 1 µg up to 20 kg (Moles *et al*., 2005). Regardless of seed size, many plants develop hard packaging structures that are most commonly derived from layers of the seed coat (testa) and pericarp (in gymnosperms, they are derived from seed coats and cones). Hard encapsulations may protect seeds either only temporarily or at all times during their life span, including during development, dispersal (especially over long distances – summarized by Nathan *et al*. (2008)), storage periods before, during, or after dispersal, and germination. Particularly in regions with irregular rainfall, the seed stage often involves diaspore heteromorphism (Arshad *et al*., 2018; Yang *et al*., 2020; Gianella *et al*., 2021), environmentally regulated dispersal (e.g. Seale & Nakayama, 2020), and extended storage periods in the canopy (termed serotiny; Lamont *et al*., 2020) or in the soil (known as dormancy; Baskin & Baskin, 2014). On the extreme end, the role of seed encapsulation is well illustrated in fire‐prone environments, where the often short‐lived seeds are protected and preserved beyond maturity inside hard, lignified fruits (or in cones in gymnosperms) that only open in response to fire (Lamont *et al*., 2020). Similarly, in physically dormant seeds, the testa or pericarp forms a sealed barrier that is often so effective that it can delay germination for years unless physically or chemically disrupted (e.g. by fire; Baskin & Baskin, 2014). In both cases, it is primarily the encapsulating tissue that protects the (mature) seed and prevents it from entering the next life stage. Therefore, it is interesting to know whether their encapsulating tissues share any common features for storage and protection. Since structural integrity of the covering layers and seed viability are also important in zoochorous interactions, we additionally explore functional aspects for scatter‐hoarding of large propagules (synzoochory; Vander Wall, 2010) and ingestion and (often intact) excretion of smaller ones by granivores (endozoochory; Costea *et al*., 2019). Motivated by the morphological and functional diversity of hard seed‐packaging structures, the aim of this review is to provide an overview of different anatomical features and properties that contribute to specific sclerenchyma functions, focusing largely on angiosperms (though some interesting examples of gymnosperms are also mentioned).

## Sclerenchyma forms hard static and responsive encapsulations

**Fig. 1:**
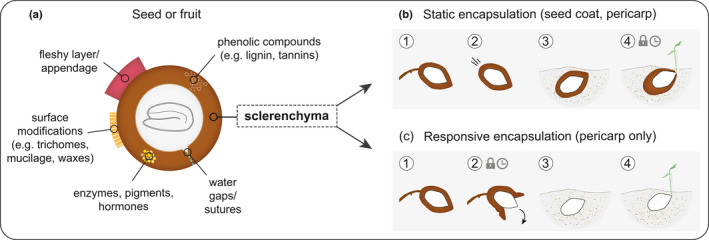
Schematic overview of the functional aspects of seed encapsulation in angiosperms. (a) Generalized, sclerenchymatous shell with common chemical and structural properties of seed coats (testa), fruit walls (pericarp), and caryopses (testa and pericarp fused). As part of the sclerenchymatous tissue, a predetermined breaking point (water gaps or sutures) and a number of multifunctional compounds (aromatic and aliphatic) are often found. In some species, the hard shell is covered by a fleshy layer (e.g. mesocarp) or has a fleshy appendage (e.g. aril). If surface structures (trichomes, mucilage, or waxes) are present, they typically regulate water interactions. Depending on the structure of sclerenchyma, encapsulations may be static or responsive. (b) Static and (c) responsive encapsulation with different states: (1) maturity, characterized by on‐plant storage of the encapsulated seed(s); (2) diaspore release from the mother plant, which involves detachment in static and opening in responsive encapsulations; (3) after dispersal, corresponding to soil storage; and (4) germination, which requires imbibition and radicle penetration through the covering layers. In seeds with physical dormancy and in serotinous fruits, stages 4 and 2, respectively, are delayed and require specific environmental cues to be ‘unlocked’ (clock and lock symbols in (b) and (c)).

Generally, seed encapsulations protect the embryo and, if present, its resources (endosperm, perisperm) against physical, chemical, and biological damage (Fig. 1a). In hard encapsulations, sclereids and fibres provide mechanical reinforcement by thickened secondary cell walls (Evert, 2006). Sclerenchyma derives etymologically from the Greek word *sklērós* (= ‘hard’), indicating that hardness is a central feature. In fruits and seeds, tissue hardening is often a result of lignification, but hard secondary cell walls may also consist mainly of polysaccharides. A good example are the hard, nonlignified endosperm cell walls in many palm seeds – for example, date palm (*Phoenix dactylifera*) or the tagua nut (*Phytelephas macrocarpa*) – which serve simultaneously as storage and protective tissues (Buckeridge, 2010). As we will demonstrate in this review, the properties of hard seed coats and pericarps depend strongly on the composition and arrangement of sclerenchyma.

Based on their performance, we classify sclerenchymatous encapsulations as either static, meaning dimensionally stable and indehiscent (nondeforming on the macroscopic level, Fig. 1b), or responsive, meaning able to deform/open macroscopically when experiencing changes in humidity or temperature, typically to release seeds (Fig. 1c). With this reductionist approach, we aim to understand the underlying principles and tissue properties rather than the molecular processes that lead to their formation. It is also noteworthy that many diaspores and fruits are obviously more complex than the scheme in Fig. 1 suggests. For example, static fruits may contain more than one seed, and each seed may again be encapsulated by a hard shell (testa), as in the Brazil nut (*Bertholletia excelsa*; Sonego *et al*., 2019) or in trample‐burrs (e.g. *Ibicella lutea*; Horbens *et al*., 2014). Furthermore, combined systems of responsive and static encapsulations exist; that is, hard‐shelled seeds inside responsive fruits, such as in the Chinese witch hazel (*Hamamelis mollis*; Poppinga *et al*., 2019), or hard‐shelled seeds (testa and pericarp fused/ caryopsis) with a responsive remnant of the carpel, known as awn (in the Geraniaceae family; Abraham & Elbaum, 2013). Awns are moisture‐responsive appendages that are attached to seeds and enable (self) dispersal and burial. We include responsive appendages as part of responsive encapsulations. However, since appendages do not fulfil the criterion of full embryo encapsulation, a more differentiated discussion is required in the following sections.

Regarding the formation of the two encapsulation types (Fig. 1b,c), there are ontogenetic differences. Whereas static encapsulations can be formed from fruit and seed tissues, responsive encapsulations usually only develop from fruit tissues. The two systems also clearly differ in their ability to protect seeds over time. Static structures, such as nutshells, provide protection for seeds during all stages – from development/maturity, during dispersal and storage, until germination (Fig. 1b). They often possess supporting structures (e.g. wings, awns, fleshy arils) that develop from the flower, fruit, seed coat, or modified leaves (bracts) to promote diaspore dispersal. Responsive encapsulations, on the other hand, typically remain attached to the plant as aerial seedbanks and are thus not dispersed (Fig. 1c), except when removed deliberately by animals (Talluto & Benkman, 2014) or accidentally by any means, or in rare cases by the plant itself via abscission (Abraham & Elbaum, 2013; Yang *et al*., 2020). Close to maturity, most responsive encapsulations either open up to release seeds (dehiscence), or remain closed until environmentally stimulated, thereby delaying seed dispersal (stage 2 in Fig. 1c) until conditions are more favourable for germination and seedling establishment. Consequently, responsive encapsulations provide protection only during seed development and on‐plant storage (short or long term). This is certainly beneficial to limit predispersal seed predation in any biome (Hulme & Benkman, 2002), but appears to be particularly advantageous in habitats where the pre and postdispersal environments differ dramatically in terms of nutrient and water availability (and presence of granivores); for example, arid and fire‐prone regions. In heterogeneous habitats, responsive encapsulations often share surprisingly similar functionalities across angiosperms and gymnosperms that can be interpreted as a result of convergent evolution (e.g. Clarke *et al*., 2013). Examples are the woody fruits in the Myrtaceae (e.g. *Eucalyptus* spp., *Melaleuca* spp.), Proteaceae (e.g. *Hakea* spp., *Banksia* spp., *Xylomelum* spp.) and woody cones in the Pinaceae (e.g. *Pinus* spp.) and Cupressaceae (e.g. *Sequoiadendron giganteum*), which may all reduce seed loss or damage from granivores, desiccation, and fire (Moya *et al*., 2008; Talluto & Benkman, 2014; Lamont *et al*., 2016, 2020).

## Water regulation is crucial for seed survival and protection

As a key element of plant life, water is not only relevant for the metabolic activity of the embryo, but it also affects the properties of the encapsulating tissue. Therefore, it is essential that encapsulations regulate the water content of both components accordingly. The effects of water regulation are well recognizable during the final step of seed development on the maternal plant: the sclerenchymatous cells that form the encapsulation (or parts of it) die and typically dry together with the embyro(s) inside. After this point, seed viability depends strongly on the hydration level. Therefore, a major function of many hard encapsulation tissues is to retain a low moisture content inside the seed. A low seed moisture content may increase the desiccation tolerance of the embryo and induces dormancy in a large number of species (Werker, 1997; Baskin & Baskin, 2014). In addition, it is well known that water acts as a plasticizer in lignocellulosic tissues. Consequently, drying increases the stiffness (resistance against deformation) of the protective layer. Seeds benefit from this metabolically independent stiffening process while they become independent from the mother plant and increasingly attractive for predators. Hard structures may thereby reduce seed detection and predation by granivores during storage phases in the canopy and in the soil. In buried seeds, reduced detection has been linked to drying‐induced dormancy, because dormant seeds fail to release sufficient concentrations of volatile metabolic by‐products that attract predators (Paulsen *et al*., 2013). Dry, woody packagings impose additional handling costs on granivores for seed extraction, which may lead to avoidance and scatter‐hoarding (Vander Wall, 2010; Lamont *et al*., 2016). To maintain a low seed moisture content or to impose physical dormancy, plants may chemically waterproof the cuticle or the tissues beneath it; for example, via impregnations with suberin (Fedi *et al*., 2017), waxes, cutin, lignin (del Rio *et al*., 2017; Landucci *et al*., 2020), quinones, callose, silica, or inclusions of oil droplets or tannins in the lumen (Baskin & Baskin, 2014). Most compounds are in fact associated with more than just one function, which is probably best illustrated in flavonoids, such as proanthocyanidins. Proanthocyanidins not only regulate the water permeability of tissues but also interfere with microbes and act as radical scavengers that likely reduce oxidative damage in soil and canopy‐stored seeds (Debeaujon *et al*., 2007; Smýkal *et al*., 2014; Huss *et al*., 2019).

In static encapsulations, waterproofing may only be beneficial until conditions are suitable for germination. As germination requires water uptake by the embryo (imbibition), all covering layers play a central regulatory role in this step (Fig. 1a). In addition to chemical compounds, physical properties, such as the presence and properties of pits, drying‐induced cracks and the overall tissue thickness determine the water permeability (Smýkal *et al*., 2014). Surface modifications, like trichomes or rapidly swellable mucilage (containing pectin, hemicelluloses, and cellulose fibrils), may additionally regulate and facilitate water adsorption, and soil substrate adhesion, and thus germination (Western, 2012; Mamut *et al*., 2014). Some of the most important features of many static encapsulations are the specialized suture tissues and water gaps that allow directed water movement towards the embryo and subsequent radicle protrusion. For species with physical dormancy, 24 different kinds of water gap regions have been reported in 16 plant families (Gama‐Arachchige *et al*., 2013). Characteristic features of water gap regions are deformable surface structures, porous tissues (e.g. tracheid bars in the hilum), a locally decreased coat thickness, and a discontinuous tissue structure lacking secondary cell wall formation and lignification (Gama‐Arachchige *et al*., 2013; Smýkal *et al*., 2014; Janská *et al*., 2019). These ‘lignification gaps’ are more readily degraded by common soil fungi, and thereby increase the water permeability of hard layers (Sperber *et al*., 2017). Taking the effectiveness of microbial attacks into account, it may not be surprising that some hard seed coats (e.g. in the Brassicaceae) store hydrolytic enzymes with antimicrobial activity, which remain functional for decades and are released upon hydration (Raviv *et al*., 2017). Furthermore, encapsulating tissues often contain phytohormones and nutrients, which promote embryo growth and radicle protrusion, among other things (reviewed by Raviv *et al*., 2018).

## Tissue anatomy drives mechanical functions

Sclerenchyma performs differently in static and responsive encapsulations due to the tissue arrangement, cell wall structure, and composition. This requires developmental control during cell differentiation and involves large changes in cell geometry, as well as a specific deposition of cell wall polymers and secondary metabolites (Höfte & Voxeur, 2017). Based on the cell types, namely fibres and/or diverse sclereid subtypes, sclerenchyma can be described as fibrous or nonfibrous. A criterion to identify fibres is a large length‐to‐width ratio (often ≥ 10). If fibres are present, their orientation with respect to each other and their wall structure are important, because both parameters induce directionality (anisotropy) in terms of the mechanical properties and hygroscopic deformations – see summaries by Gibson (2012) and Eder *et al*. (2020). Broadly speaking, plants exploit fibre anisotropy for functional control, particularly for force generation. Consequently, we find fibrous tissues predominantly in responsive systems, whereas tissues in static encapsulations show a broader spectrum from entirely fibrous to nonfibrous.

### Static systems

**Fig. 2:**
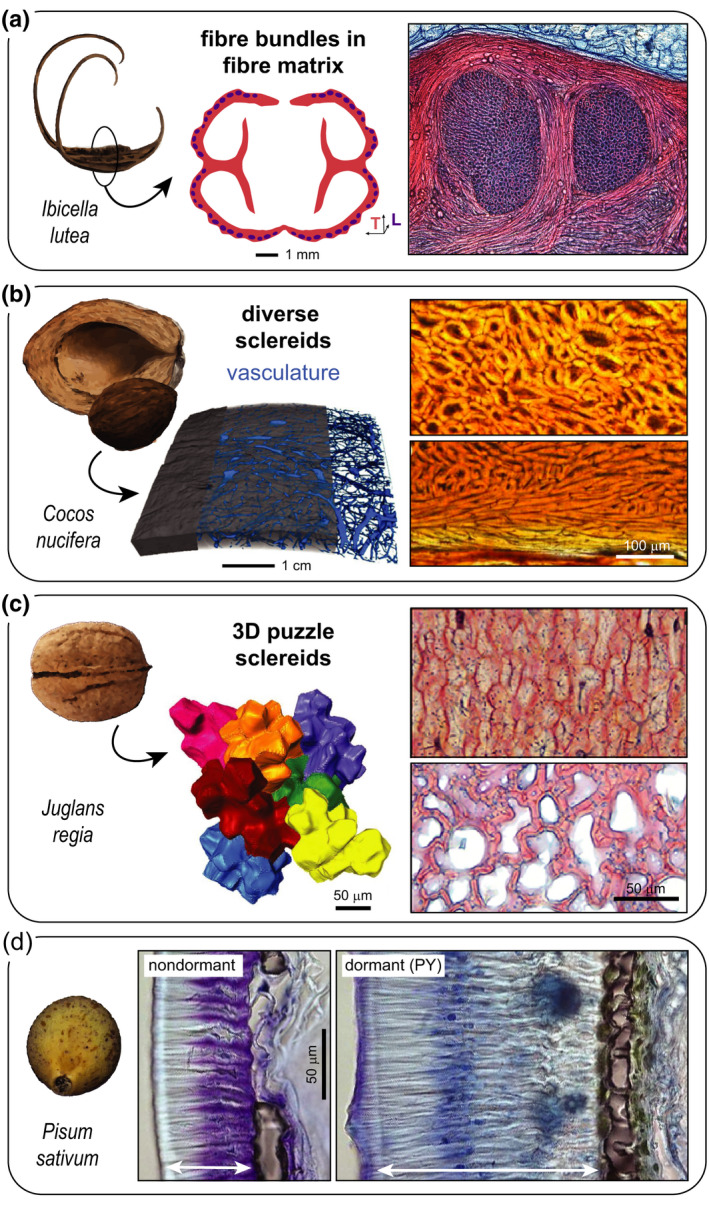
Static encapsulations and their tissue anatomy in selected species. (a) The fruit capsule (lignified endocarp) of the trample‐burr *Ibicella lutea* is entirely fibrous, containing longitudinal (L) fibre bundles in a matrix of transversely (T) oriented fibres (cross‐section inspired by Horbens *et al*. (2014); microscopy image kindly provided by Christoph Neinhuis/Institut für Botanik, TU Dresden, Germany). (b) Diverse sclereids without a preferred orientation (and vasculature) form the endocarp shell of the coconut *Cocos nucifera* (computed tomography (CT) and microscopy images adapted from Schmier *et al*. (2020a); CC‐BY 4.0). (c) The hard pericarp of the Persian walnut *Juglans regia* (cv Geisenheim120) consists of a single cell type, characterized by interlocked three‐dimensional puzzle‐shaped sclereids with thinner cell walls in the inner shell part (CT and microscopy images adapted from Antreich *et al*. (2019) and Xiao *et al*. (2020); both CC‐BY 4.0). (d) Strong differences in the testa of the pea *Pisum sativum* explain physical dormancy (PY) in wild peas (*P. sativum* subsp. *elatius* JI64) when compared with domesticated ones (cv Cameor). Notably, cuticular modifications, a thicker macrosclereid/palisade layer (indicated by arrow), and a higher content of proanthocyanidins in this layer contribute to PY. Osteosclereids form a thin inner shell layer that is well visible in the dormant wild‐type due to the surrounding greenish compounds (images adapted from Smýkal *et al*. (2014); CC‐BY 4.0). Scale bars apply to both images always. Stainings: (a, c) fuchsin–chrysoidin–astrablue; (d) toluidine blue; (b) none.

The endocarp of the devil’s claw (*Ibicella lutea* and *Proboscidea louisianica*) represents a unique adaptation for epizoochorous dispersal via trample‐burrs. The tissue is entirely fibrous (Fig. 2a) and, after incomplete initial dehiscence, it can be considered as a static system, because it fails to release seeds autonomously (Horbens *et al*., 2014). By incorporating fibres both in the transverse (network‐like) and longitudinal direction (as bundles) of the capsule and its curved extensions, the tissue can accommodate high bending, compression and torsional forces, and limit buckling when heavy mammals step on them (Horbens *et al*., 2014, 2015). The coconut endocarp (*Cocos nucifera*; Fig. 2b), by contrast, is a mixed tissue, containing both fibres and sclereids (besides vasculature) (Gludovatz *et al*., 2017; Schmier *et al*., 2020a). However, the lignified sclereids and fibres are arranged without a consistent directional pattern, which results in a static shell with a high toughness (energy absorption) and high compressive strength (Schmier *et al*., 2020b). Based on the colouring of the unstained tissue (Fig. 2b), the cell walls also appear to be heavily impregnated (presumably with some flavonoids, in addition to stilbenolignins; del Rio *et al*., 2017). A similar tissue arrangement (Table 1) along with a high lignin content occurs in many other (tropical) species (Landucci *et al*., 2020); for example, in the seed coat of macadamia (*Macadamia integrifolia*; Schüler *et al*., 2014), the endocarp of the cocoyol fruit (*Acrocomia mexicana*; Flores‐Johnson *et al*., 2018), and in the mesocarp of the Brazil nut (*B. excelsa*; Sonego *et al*., 2019), though fibres are more bundled and form network‐like structures in the latter. There are also nonfibrous, mechanically strong, static encapsulation tissues. These may consist of thick‐walled sclereid cells, such as the interlocking three‐dimensional (3D) puzzle sclereids in walnut shells (*Juglans regia*; Fig. 2c), which are arranged with density and lignin gradients (Antreich *et al*., 2019; Xiao *et al*., 2020). Gradient structures are known to increase a material’s toughness by gradually matching layers with different mechanical properties (Naleway *et al*., 2015), which improves load transfer and thereby potentially delays crack formation and delamination. As a rule of thumb, strength (defined as the maximum force per cross‐sectional area) and stiffness increase with density in lignocellulosic tissues (Gibson, 2012). One way to increase the strength (and often stiffness) is thus to increase the cross‐sectional area by thickening of the secondary cell wall. Another way is the development of undulating cell walls that also interlock neighbouring cells (as in walnuts and pistachios). This may additionally prevent global failure once the middle lamella ruptures (Huss *et al*., 2020). The middle lamella connects adjacent cell walls and is initially pectin rich, but it then starts to lignify as secondary wall formation progresses (Donaldson, 2001; Xiao *et al*., 2020). In comparison with cell walls, a detailed understanding of the mechanical properties of the middle lamella is currently still generally lacking owing to its nanoscale size – see summary by Zamil & Geitmann (2017). However, the numbers provided by Gibson (2012) might serve as a rough orientation, with a reported (tensile) strength of 25–75 MPa for lignin and *c. *100–600 MPa for wood cell walls, pointing to a weaker middle lamella.

**Table 1:** Overview of the anatomical diversity in static and responsive seed encapsulations (and appendages) on different levels, including the tissue arrangement, the cell types employed, and the developmental origin (organ part). Functional features related to seed protection and dispersal are highlighted in different taxa.

Type	Tissue arrangement	Cell types	Organ part	Taxon	Functional features	References
Static shell	Uniform	Isodiametric sclereids	Seed coat	*Pinus koraiensis*	?	Antreich *et al*. (2019); Huss *et al*. (2020)
		3D puzzle sclereids	Endocarp	*Pistacia vera*	Tensile strength, toughness, interlocking	Huss *et al*. (2020)
	Uniform with density gradient	3D puzzle sclereids	Pericarp	*Juglans regia*	Tensile + compression strength, interlocking	Antreich *et al*. (2019); Xiao *et al*. (2020)
	Layers	Macrosclereids, osteosclereids	Seed coat	Fabaceae	Waterproofing, PY	Smýkal *et al*. (2014); Janská *et al*. (2019)
		Macrosclereids, other sclereids	Seed coat + pericarp	*Centaurea jacea*, *Centaurea scabiosa*	?	Bobrov & Romanov (2019)
		Macrosclereids (endo), parenchyma	Pericarp	*Calycanthus* spp., *Hernandia nymphaefolia*, *Umbellularia californica*, *Cassytha* sp.	?	Romanov *et al*. (2018); Bobrov & Romanov (2019)
	Network‐like	Fibre bundles surrounded by fibre matrix	Endocarp	*Ibicella lutea*, *Proboscidea louisianica*	Compression + bending strength, hoof attachment	Horbens *et al*. (2014); Horbens *et al*. (2015)
	Fibre‐reinforced matrix	Fibres/bundles (unspecific orientation), embedded in sclereids	Endocarp	*Cocos nucifera*	Compression strength, waterproofing	Gludovatz *et al*. (2017); Schmier *et al*. (2020)
			Endocarp	*Acrocomia mexicana*	Compression strength, hardness	Flores‐Johnson *et al*. (2018)
			Mesocarp	*Bertholletia excelsa*	Compression strength, impact resistance	Sonego *et al*. (2019)
			Seed coat	*Macadamia integrifolia*	Compression strength	Schüler *et al*. (2014); Huss *et al*. (2020)
Responsive valves	Uniform	Cellulosic keel cells	Pericarp	*Delosperma nukerense*	Hygro‐responsive unfolding (wetting)	Harrington *et al*. (2011)
	Bilayer	Fibres and sclereids with different MFAs in two layers (30° and 74°)	Cone scale	*Pinus radiata*, *Pinus halepensis*	Thermo, hygro‐responsive bending (drying, fire), serotiny	Dawson *et al*. (1997); Moya *et al*. (2008)
		Parallel fibres (high MFA) with 90° shift between two layers	Pericarp	*Bauhinia variegatae*	Hygro‐responsive twisting (drying)	Armon *et al*. (2011)
		Parenchyma (meso) and two fibrous layers with 90° shift (endo)	Pericarp	*Sesamum indicum*	Hygro‐responsive bending (drying)	Shtein *et al*. (2016)
		Fleshy (exo) and fibrous layer (endo)	Pericarp	*Hamamelis mollis*	Hygro‐responsive bending + tightening (drying), ballistic seed ejection	Poppinga *et al*. (2019)
	Bilayer with network	Branched fibre bundles (meso, high MFA) and parallel fibres (endo, low MFA)	Pericarp	*Banksia attenuata*	Thermo, hygro‐responsive bending (drying, fire), serotiny	Huss *et al*. (2018)
Responsive appendage	Bilayer	Parallel fibres with different MFAs in two layers	Awn (mericarp)	*Erodium* sp., *Geranium* sp., *Pelargonium* sp.	Hygro‐responsive twisting/‌coiling/‌bending (drying), ballistic ejection	Evangelista *et al*. (2011); Abraham & Elbaum (2013)

3D, three‐dimensional; exo, exocarp; meso, mesocarp; endo, endocarp; MFA, microfibril angle; PY, physical dormancy.

In terms of cell types, a surprisingly uniform arrangement of isodiametric brachysclereids can be found in the seed coat of the Korean pine (*Pinus koraiensis*; Antreich *et al*., 2019). In comparison with hard coats in angiosperms, this tissue appears less differentiated (Table 1); yet, in terms of strength, it can compete with some angiosperm shells (Huss *et al*., 2020). Generally, it seems that thick shells rarely consist of a single cell type – most static shells show various cell types, often arranged in layers. Characteristic sclereid layers are typical for legume seed coats (Smýkal *et al*., 2014): the outer (palisade) layer consists of elongated and tightly packed macrosclereids (Fig. 2d), followed by a layer of osteosclereids (and loose parenchyma). As Smýkal *et al*. (2014) reported, the thickness of the palisade layer and its impregnation with proanthocyanidins are crucial factors that contribute to physical dormancy in many seeds of the Fabaceae family (Fig. 2d). The parallel arrangement of macrosclereids, perpendicular to the surface, is curious from a mechanical point of view, because it seems prone to crack formation along the lumen and middle lamella. Nonetheless, this arrangement of macrosclereids occurs in static shells across many plant families (e.g. Romanov *et al*., 2018; Bobrov & Romanov, 2019). However, no detailed mechanical characterization has been performed to date. As indicated by Bobrov & Romanov (2019), there are many other possible arrangements of sclerenchymatous tissues in static shells (see also Table 1), which mostly involve the cell types that we previously mentioned. However, many encapsulating tissues still remain unstudied to date and may contain other sclereid types, and compounds that we have not discussed (e.g. calcium oxalate crystals).

### Responsive systems

**Fig. 3:**
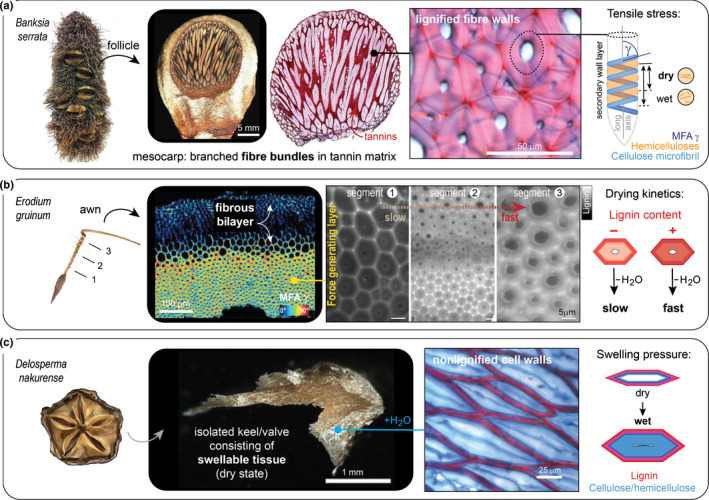
Responsive encapsulations and their tissue anatomy in selected species. (a) In the mesocarp of banksia (*Banksia serrata*) follicles, forces are generated by a network of branched fibre bundles surrounded by a matrix of condensed tannins (microscopy images kindly provided by Friedrich Reppe, MPI of Colloids and Interfaces). The bundles are lignified (fuchsin–chrysoidin–astrablue (FCA) staining) and, as illustrated schematically, tensile stresses are generated by drying and shrinkage of the hemicelluloses in the secondary cell wall, which are surrounding cellulose microfibrils that form a large angle (microfibril angle (MFA) *γ* = 75–90°) with the longitudinal fibre axis. This shrinkage results in shortening of fibres upon drying (Huss *et al*., 2018). (b) Force generation in awns of the storksbill *Erodium gruinum* occurs in a similar manner, but fibres (with a large MFA and a tilted helix) are arranged parallel to each other instead of in bundles, and drying kinetics are modulated by a lignin (ferulic acid) gradient. Polarization microscopy image adapted by permission from RightsLink: John Wiley and Sons *New Phytologis*t (Abraham & Elbaum, 2013) © 2013, and autofluorescence images from RightsLink: Springer Nature *Cellulose* (Abraham *et al*., 2018) © 2018. (c) Seed pods of the iceplant *Delosperma nakurense* open when wetted with liquid water due to the swelling pressure of cellulosic keel cells (microscopy images adapted by permission from RightsLink: Springer Nature *Nature Communications* (Harrington *et al*., 2011) © 2011). Tissue is stained with FCA, showing nonlignified cell walls that are able to absorb large amounts of water and thereby exert swelling pressure.

Responsive encapsulations typically rely on valves that deform when exposed to wetting, drying, and/or heating. Discontinuous tissue structures serve as predetermined breaking points (sutures) for separation and are often simply realized by means of a locally reduced tissue and cell wall thickness, and/or lignification gaps (Mummenhoff *et al*., 2009; Huss *et al*., 2018). However, some sutures are more sophisticated, as in *Banksia* spp. follicles, which contain a layer of waxes between two valves, which melt at high ambient temperatures, thereby facilitating opening (e.g. Huss *et al*., 2018). In all responsive systems of this review, force generation largely arises from swelling or shrinkage of carbohydrates in the secondary cell wall of fibres or elongated sclereids (Fig. 3). Owing to the molecular structure, branched polysaccharides (hemicelluloses) strongly interact with water molecules, whereas linear polysaccharides (cellulose) only weakly interact with water due to their high crystallinity (Höfte & Voxeur, 2017). As stiff, bundled polymer chains, known as microfibrils, cellulose can direct moisture‐dependent volume changes and modulate the mechanical properties (notably strength and stiffness) of the entire fibre and fibrous tissues (summarized by Eder *et al*. (2020)). This is achieved by varying the angle of cellulose microfibrils in the secondary cell wall with respect to the fibre long axis (scheme in Fig. 3a), known as the microfibril angle (MFA). Fibres with a large MFA are more elastic and undergo stronger hygroscopic length changes. Further fine‐tuning can be achieved by varying the content of aromatic moieties/lignin: fibres with a high lignin content are stiffer (Köhler & Spatz, 2002) and dry faster than those with a lower lignin content (Abraham *et al*., 2018). These properties make fibres versatile elements in sclerenchymatous tissues: in the shape of a branched network of bundles (Fig. 3a), for example, lignified fibres with a large MFA (in the mesocarp) generate the required tensile stresses for the opening of *Banksia* sp. follicles upon drying (Huss *et al*., 2018). Shortening of the entire branched network is, however, restricted by the adjacent endocarp layer, consisting of parallel fibres with a small MFA (close to 0°). Bending of the valves occurs at high temperatures due to a temperature‐dependent reduction of the endocarp stiffness, which allows to release the accumulated stresses in the mesocarp. Without the temperature dependency, this so‐called bilayer arrangement is typical for drying‐induced opening movements and can be found in many unrelated species with minor modifications. For example, a 90° shift between two layers of parallel fibres (with large MFAs) results in helical deformations upon drying, as shown in seed pods of orchid trees (*Bauhinia variegate*; Armon *et al*., 2011). In a similar manner, the responsive appendages (awns) of some grasses, such as wheat (*Triticum* sp.; Elbaum *et al*., 2007), storksbill (*Erodium* sp.; Evangelista *et al*., 2011), or cranesbill (*Geranium* sp.; Abraham & Elbaum, 2013), exploit changes in relative humidity for dispersal and self‐burial of seeds. The mechanism in awns is mostly based on parallel fibres that show a large MFA (or random orientation; Elbaum *et al*., 2007) in the stress‐generating layer and a small MFA (≤ 30°) in the other layer (Fig. 3b). If the cellulose helix is tilted, awns are coiling instead of bending (Abraham & Elbaum, 2013). This applies to *Erodium gruinum* awns (Fig. 3b) that also regulate the drying kinetics by means of a lignin (ferulic acid) gradient, which results in faster drying of the top segment in comparison with the base, where the seed is attached (Abraham *et al*., 2018). By definition, hygroscopic materials (i.e. cell walls) respond not only to drying but also to wetting, as illustrated by the keel tissue in the seed capsule of the ice plant *Delosperma nakurense* (Harrington *et al*., 2011). Though the detailed composition of the swellable ‘cellulosic’ wall layer and its water interactions remain to be studied in depth, it is known that its swelling pressure (Fig. 3c) causes the flexing movement (valve unfolding) within minutes after wetting. In the context of different plant movements, the role of water and its swelling pressure have been well reviewed by Dumais & Forterre (2012) and will therefore not be discussed further.

## Adaptive solutions

Morphological changes of hard packaging structures (e.g. shell thickness, size, or shape) have far‐reaching consequences for dispersal, protection, and germination. They may occur within the lifetime of an individual (Arshad *et al*., 2018) or over evolutionarily relevant timescales (Smýkal *et al*., 2014), both in static and responsive encapsulations. A typical example in static systems is the strategic development of varying seed sizes and diaspore morphs by the same individual of stonecresses (*Aethionema arabicum*), which consequently show different modes of dispersal and different levels of physical dormancy (Arshad *et al*., 2018). In different populations of the candlestick banksia (*Banksia attenuata*), plants develop follicles (responsive systems) with different valve curvatures along a climatic gradient, which results in higher levels of serotiny and higher opening temperatures in drier regions (Huss *et al*., 2018). These morphological adaptations and the corresponding differences in physical dormancy and serotiny are often interpreted as bet‐hedging strategies to maximize survival in unpredictable environments (Clarke *et al*., 2013; Arshad *et al*., 2018; Yang *et al*., 2020; Gianella *et al*., 2021). In bet‐hedging species, dormancy appears to be linked to a higher polyphenol content in seed coats, resulting in darker morphs (Gianella *et al*., 2021). In the barrelclover *Medicago truncatula* (Fabaceae), four genes related to the flavonoid metabolism and seven peroxidases and thio/peroxiredoxins have been associated with differential dormancy along an aridity gradient (Renzi *et al*., 2020). However, many of the underlying developmental differences remain largely unstudied on the tissue level.

**Fig. 4:**
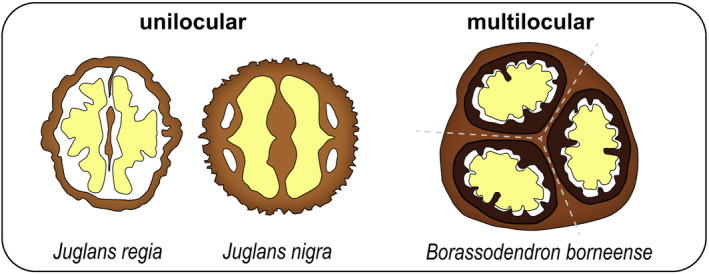
Mechanical reinforcement by septa and ingrowths in uni‐ and multilocular fruits (schematic cross‐sections). The Persian walnut *Juglans regia* and the eastern black walnut *Juglans nigra* each develop one locule with one seed (yellow), partially separated by a lignified central septum. The septum is much thicker in *J. nigra*, which significantly reinforces the shell from within by increasing the cross‐sectional area for load bearing. A similar effect is achieved by the local shell ingrowths (dark brown) in the lignified, multilocular fruits of the Borneo giant fan palm *Borassodendron borneense* (developing three locules separated by septa; marked by dashed lines. Drawing inspired by Bellot *et al*. (2020). Lignified septa and local shell ingrowths can be found in many species of the walnut family (Juglandaceae) and the palm family (Arecaceae).

Potential strategies to increase the mechanical resistance of static encapsulations can be derived from seeds with strong animal interactions (zoochory). Shell geometry is particularly important to resist shell cracking by granivores via compression loading, for example. Owing to a higher rigidity, small spherical shells are more advantageous than elongated shells of similar size and thickness (Huss *et al*., 2020). For intact (avian) gut passage, geometry seems to be irrelevant, as long as the encapsulated seeds are small enough for ingestion. Surprisingly, a single layer of lignified cells is often already sufficient for protection in the digestive tract (e.g. 51% survival in the Hungarian milkvetch *Astragalus contortuplicatus*; Costea *et al*., 2019). In large diaspores, an important aspect of mechanical resistance (besides shell geometry and thickness) is the development of thick, lignified septa and ingrowths in uni and multilocular fruits. Fig. 4 shows that the internal shell structure differs strongly in the fruits of the closely related Persian walnut (*J. regia*) and eastern black walnut (*J. nigra*). In both species, the seed is surrounded by an ellipsoidal hard shell and partly separated by a septum. However, the septum in *J. regia* is thin and mechanically insignificant, whereas in *J. nigra* it is extremely thick and reinforces the shell internally (‘cross‐struts’; Fig. 4). Lignified ingrowths and partitioning of the locule do not occur only in the Juglandaceae (*Juglans* spp., *Carya* spp.). Interestingly, they also occur in many species of the palm family (Arecaceae); for example, in the Borneo giant fan palm (*Borassodendron borneense*, Fig. 4), the coco de mer (*Lodoicea maldivica*), the Bismarck palm (*Bismarckia nobilis*), and in Eugeissona palms (*Eugeissona* spp.) (Bobrov *et al*., 2012; Bobrov & Romanov, 2019; Bellot *et al*., 2020). In these species, the local shell ingrowths increase the cross‐sectional area for load bearing and the resistance against bending. Therefore, locule partitioning (by septa) and the development of local shell ingrowths are effective strategies for mechanical reinforcement of static encapsulations. It seems that this strategy evolved independently in various taxa with large seeds and animal interactions.

## Conclusions and perspectives

Hard encapsulations for seed protection and dispersal are morphologically extremely diverse. On a general basis, the functionality of packaging structures arises from characteristic modifications of the tissues surrounding the embryo. Despite large genetic and ecological differences, many encapsulations share rather common features, such as predetermined breaking points, employment of fibres for force generation, and the incorporation of flavonoids for long‐term storage and protection. However, many questions still remain open. In the context of cell shapes, for example, it is unclear why 3D puzzle sclereids are rather rare in static structures despite their high strength (Huss *et al*., 2020). Moreover, to what extent can plants adapt their seed encapsulations to environmental changes? And could some of these principles or materials (e.g. chemicals or nutshell residues) be exploited to develop artificial seed coats for conservation, considering that seed coating is already common practice in industry, even though it is currently highly adapted for agricultural usage (Pedrini *et al*., 2017)? So far, interdisciplinary approaches have been promising in the study of the relationship between structure and function in seed encapsulations, particularly by following developmental changes of tissues, their composition, and mechanics (e.g. Horbens *et al*., 2014, 2015; Antreich *et al*., 2019; Xiao *et al*., 2020). Future studies of seed packaging structures have the potential to broaden our understanding in many fields, ranging from molecular processes during cell wall formation to ecological interactions and evolutionary adaptations, as well as biomimetic applications and 3D printing of lignocellulosic shells.
